# More than a meeting: identifying the needs of the community-based seniors’ services sector as providers of health promotion services

**DOI:** 10.1186/s13104-021-05753-y

**Published:** 2021-08-30

**Authors:** Catherine E. Tong, Joanie Sims-Gould, Sarah Lusina-Furst, Heather McKay

**Affiliations:** 1 Centre for Hip Health & Mobility, University of British Columbia, Robert H.N. Ho Building, 7/F - 2635 Laurel St., Vancouver, BC V5Z 1M9 Canada; 2 School of Public Health & Health Systems, Faculty of Health, University of Waterloo, Waterloo, Canada; 3 Department of Family Practice, Faculty of Medicine, University of British Columbia, Vancouver, Canada

**Keywords:** Health services, Older adults, Home and community-based care and services, Conference, Evaluation

## Abstract

**Objective:**

Many economically developed countries have seen a decline in publicly funded community programming. Within this context, community-based seniors’ service (CBSS) organizations have been increasingly tasked to deliver programs to support the health and wellbeing of older citizens (e.g., home support, physical activity programs, and chronic disease management education). The primary objective of this study was to capture of the current needs of CBSS leaders in British Columbia, Canada, who attended a seminal event in the CBSS sector’s development—the inaugural Summit on Aging.

**Results:**

Our evaluation of the Summit included: pre/post Summit surveys (N = 79/76), ethnographic observations, and follow-up interviews (n = 22). Our detailed evaluation plan may inform others undertaking similar data collection; the most informative results were derived from the follow-up interviews and our findings suggest that interviews may be sufficient for similar evaluations. Summit delegates identified key opportunities to strengthen the CBSS as a sector, including enhanced collaboration; improved mechanisms that foster connecting and collaborating; and more resources, including training and qualified staff, to increase their capacity to deliver community-based health services. These findings echo work already completed in the community-based health promotion sector.

**Supplementary Information:**

The online version contains supplementary material available at 10.1186/s13104-021-05753-y.

## Introduction

Through neoliberal reforms, a decline in the welfare state [1], and austerity measures [2], many economically developed countries have seen a decline in comprehensive, publicly funded support services for older adults [3]. Due to the hollowing-out of centrally supported services [4], greater demands have been placed on community-based seniors’ service organizations (CBSSs) operated primarily by non-governmental organizations (NGOs), which are increasingly tasked with delivering health promotion programming [5–7]—a task formally outside the scope, expertise, and capacity of many organizations.

To support CBSSs as they increasingly provide health services to older community members, the inaugural Provincial Summit on Aging (herein referred to as ‘the Summit’) was designed and held in British Columbia on November 2nd and 3rd, 2017. The primary objective of the Summit was to gather CBSS leaders to understand their needs and develop a plan of action that would allow them to provide community-based health services (e.g., home support, physical activity programming, chronic disease management programs).

The aim of this research was to systematically identify the needs and collaborative actions of the CBSS, who participated in the inaugural Summit as providers of health care services to older adults.

## Main text

### Research design and methods

We implemented a mixed-method evaluation approach, which included: a pre- and post-event online survey; thematic notes from breakout sessions, large group sessions, and ethnographic observations; and a 6-month post-event semi-structured telephone interview. We received ethics approval from University of British Columbia’s Behaviours Research Ethics Board for the evaluation of the Summit.

#### Overview of the Summit

The Summit occurred over two full days. Delegates included 198 CBSS leaders, older adult community members, and our research team. Keynote presentations and panel dialogues addressed demographic trends, a growing need for partnerships and collaboration, emergent opportunities to strengthen the CBSS, and service trends. Break out sessions were designed to promote knowledge sharing amongst delegates. For detailed proceedings, see http://www.seniorsraisingtheprofile.ca/gatherings/provincial-summit.

#### Pre- and post-event online survey

Online survey invitations were sent by email to all registered delegates (n = 216 for the pro-conference survey, n = 198 for the post-conference survey). Delegates self-selected to participate and consent was obtained at the beginning of each survey. We received 79 responses to the pre-Summit survey, which asked five questions focused on demographics and expectations leading into the Summit. We received 76 responses to the post-Summit survey, which asked eight questions focused on learnings and impact. Survey data are presented in Table 1 (see Additional file 1).

**Table 1:** Pre-Summit (n = 79) and post-Summit (n = 76) surveys

Pre-Summit survey (n = 79)	
Variables	n (%)
Type of organization	
Not for profit	55 (73.3)
Local government	13 (17.3)
Health services (including local health authority staff)	5 (6.7)
Provincial government	5 (6.7)
Academic institution	5 (6.7)
Health professional	3 (4.0)
Funding agency	1 (1.3)
Other	12 (16.0)
What do you hope to take away from the Summit?	
Seeking partnerships & collaborations	41 (51.8)
Gain new knowledge	28 (35.5)
Meet with old colleagues, deepen established relationships	10 (12.7)

Post-Summit survey (n = 76)	
Was the summit valuable to you?	
Yes, very valuable	30 (39.5)
Yes, somewhat valuable	38 (50.0)
No, not valuable	5 (6.6)
Not applicable	3 (3.9)
What are the most important next steps for Summit organizers?^a^	
Development of a funding strategy	23 (33.3)
Support for community-level planning and coordination	22 (31.9)
Launch of an online knowledge sharing platform	15 (21.8)
Other needs	9 (13.0)

^a^Missing data, n = 69 for this question

#### Thematic notes and ethnographic observations

Active Aging Research Team (AART) staff and graduate students trained in note-taking and ethnographic observations [8] were assigned to specific Summit sessions. We co-created ‘observation guides’ to assist with the process and ensure consistency; notes and observations were digitally transcribed.

#### Post-Summit interviews

The post-Summit interviews occurred approximately 6 months after the Summit and consisted of the completion of an online form and a semi-structured telephone interview.

*Sample* At the Summit, AART members staffed an information table to recruit interview participants. Of the 198 delegates in attendance, 45 indicated interest in participating. Of the 45 individuals who indicated interest and provided contact information, 30 delegates were randomly selected using a randomization tool and then contacted to confirm their interest and availability. We conducted 22 interviews. Interviewees included executive directors (n = 8), management and administrative staff (n = 8), representatives from boards and advisory committees (n = 4), and others (n = 2) involved in the provision of community-based health care services within in the CBSS.

*Data collection* Prior to the interview, participants were sent an email with a link to complete an online form. The form consisted of sixteen questions on demographics, knowledge about Summit follow-up initiatives, and their perceived facilitators and barriers facing the CBSS. Twenty participants completed the survey. Two trained research assistants conducted the interviews via telephone. The telephone interviews lasted from 10 to 27 min and were recorded. We intentionally designed the interviews to be comprehensive yet brief, building on the online form they completed. Interviews were transcribed verbatim. (See Additional file 1).

#### Consolidated analysis

Qualitative data were entered into NVivo 11. Upon completion of an initial thematic analysis for each data type, we cross-referenced high-level themes from all data sources [9, 10]: the survey responses (both quantitative and qualitative), ethnographic observations and interview transcripts. We utilized framework analysis for our consolidated analysis of all data sources [11, 12]. Initial codes included: most valuable thing about summit, partnerships and collaboration, build and strengthen CBSS sector, future meetings, sector as a space for health promotion, and opportunities. A preliminary summary of the evaluation was shared with every participant, via a plain-language report.

## Results

We identified three high-level themes that capture the needs of the CBSS: (1) the CBSS is pursuing *collaborations*, particularly with the health services, the provincial government, and with other CBSS organizations; (2) the sector seeks mechanisms to enhance *knowledge sharing and connections* across CBSS organizations; and, (3) organizations require the *capacity* to deliver these essential services, namely sustainable funding. The only salient theme that we coded for, and that did not necessarily fit into these high-level themes, was the need for transportation to CBSS programming.

### Need and response 1: the CBSS is pursuing collaborations

*Collaborations for health* Delegates recognize that they are increasingly delivering programs and services support health, both in a maintenance and preventive function. As a result, delegates would like to cultivate more formal, reliable collaborations with health care professionals, the local health authorities (British Columbia’s publicly provided health system organizations) and the provincial government—and in doing so leverage skills, resources and supports to be more effective in their health promotion programming efforts. The desired collaborations were viewed as difficult to pursue and achieve. At the time of the post-Summit interviews, new collaborations with health system players had yet to develop. One delegate reflected on their role in protecting older adults’ health in the current, siloed model:

*The opportunities are many. CBSS have the ability to reduce strain on health systems by offering community-based services that improve seniors’ health and reduce social isolation. It would help if health services recognized the value of these programs and supported them.* (data source: pre-Summit survey)

Delegates are seeking connections with local health authorities for many reasons: they are potential source of expertise and funding and the health authorities and CBSS could and should align strategic goals, activities and priorities for the provision of health supports for older adults.

### Need and response 2: the CBSS is actively seeking connections

One of the most dominant themes to come out of the Summit was a call for creating an accessible, web-based platform that would allow CBSSs from across the province to share events and programs, developments in the sector, best practices, funding opportunities, etc. A delegate reflected on why such a platform would be helpful:

*And I think that’s really great for coordinators of the seniors’ programs because then they have camaraderie between other people who are providing the same type of programming. And that’s not always possible to do on a local level because there aren’t other people providing similar programming.* (data source: post-Summit interview)

This online portal is not simply an extension of the Summit; beyond connecting leaders within the CBSS, the intent of this portal is to share lessons, co-develop resources, coordinate advocacy work, and, crucially, ensure that different members are not “*reinventing the wheel*” in their efforts to improve programming for older adults. Post-summit interviews indicate that the portal has been established and is in use (see, also, the Epilogue).

### Need 3: the CBSS requires capacity to enact meaningful and sustained change

Delegates highlighted that to execute their vital work for the burgeoning older adult population, they require the capacity to do so. Capacity comes in the form of sustained and predictable funding models; appropriate and accessible spaces; and sufficient human resources, including qualified staff. Delegates emphasized that they require sustained funding, because many of their programs are designed to be low-barrier (i.e., free or low-cost). In the delivery of community-based programming for older adults, CBSSs also require appropriate spaces and qualified staff. One delegate explained:

*I would like for somebody to convince powers-that-be that volunteers for some of these activities are simply not appropriate. That we’re putting far too much of an expectation level on volunteers to be there and to commit to regular schedules and commit to levels of responsibility and liability for which they’re really not prepared or equipped. So we need the funding to have the people in place.* (data source: post-Summit interview)

To provide preventive and supportive services for older adults, CBSSs are seeking appropriate funding models, and the right spaces and staff in place. Funders and policy makers have not yet responded to these expressed needs. These are the reflections of 198 delegates, leaders of their organizations with decades of experience, and are acutely aware of budget constraints and the needs to their local communities. Their reflections merit consideration.

## Discussion

Recent research has demonstrated that conferences and summits can be effective tools for supporting individuals, communities and organizations that provide health promotion activities [13–16]. Similar to Fox et al. [13] and Pelletier et al. [16] we too found that thoughtfully enacted and participatory conferences can foster stronger collaborations and professional networking amongst health promotion professionals, researchers and other stakeholders who otherwise do not get an opportunity to regularly meet. Launching and evaluating the Summit required more than a year of consistent planning, consultations and follow-up research. This investment of time and resources produced a province-wide event, fostered strategic linkages between organizations providing similar services to older adults, served as a launch-pad for formally recognizing this groups as a sector, and generated specific and actionable solutions to strengthen this vital work (e.g., an online communication and knowledge sharing portal). In this sense, the Summit was more than a meeting: participants moved beyond the identification of issues and used the Summit to implement change and take action. We have been able to track this action because of our evaluation efforts, in particular the follow-up interviews. While surveys were useful in characterizing participants, and observations provided our team with feedback for future Summits, the most impactful discussions around the complex, and political, nature of CBSS work was more readily captured in brief, semi-structured interviews.

### Conclusion

In 1835, the French political scientist Alexis de Tocqueville observed that the “science of association is the mother of all science” [17]. Nearly two centuries later, organizations are still trying to figure out ways to optimally work together, both within and across sectors [18]. In the context of aging societies and a declining welfare state, the work of the CBSS has taken on an increasingly important role as providers of health services, education and programming for older adults. Here we offer a general model for assessing efforts in sector development through gatherings (i.e. Summits, conferences) and have identified key areas for strengthening this growing and increasingly vital sector. The richest and most informative findings were derived from the in-depth follow-up interviews, not necessarily the observations or surveys; for others seeking to evaluate similar gatherings and collaborative efforts, interviews may be sufficient.

### Epilogue

A follow up Summit occurred in 2019 (350 attendees), and another is planned for 2022. The main outcome of the Summits has been increased connectedness and cohesiveness in the CBSS—from small, community oriented non-profit service providers to local governments and health authorities. Further, the CBSS is now better connected to the BC Ministry of Health. Through the Summits, a provincial funders table, a leadership table, and a best practice hub were established; future meetings will focus on sustaining and building on these achievements.

### Limitations

We recognize that this is a descriptive analysis conducted at the inception of the Summit and initial efforts to consolidate the CBSS sector. Our findings are not necessarily novel, and echo work completed by others [13–16]. We also recognize that this study is limited to one Canadian province, and only represents those individuals who chose and were able to participate it the Summit.

## Supplementary Information

**Additional file 1.** Surveys & Interview Guide.

## Data Availability

Additional data could be made available, upon reasonable request, by emailing Dr. Joanie Sims-Gould at simsg@mail.ubc.ca.

## Abbreviations

CBSS: Community-based seniors’ services
NGOs: Non-governmental organizations
