# Supplementary material for: More than a meeting: identifying the needs of the community-based seniors’ services sector as providers of health promotion services
Source: BMC Res Notes. 2021 Aug 30;14:339. doi: 10.1186/s13104-021-05753-y (PMC8404247; doi:10.1186/s13104-021-05753-y)
Supplement: Supplementary file 1 — Additional file 1. Surveys & Interview Guide. [file 13104_2021_5753_MOESM1_ESM.docx]

PRE-SUMMIT SURVEY

# FOR SUMMIT ON AGING ATTENDEES

*Please take time to answer the following survey questions prior to attending the Summit on Aging.*

1. In a sentence or two, tell us why you are coming to the Summit. What interests you most about the Summit event?
2. What do you hope to get out of participation in the event?
   1. Knowledge sharing – learn from the experience of others and take that information back to my work
   2. Connections – meet others working within the sector and look for ways to stay connected on areas of mutual interest
   3. Collaborations – meet others who are able to help you move forward with a specific project within the CBSS
3. In which areas do you believe partnerships with other organizations will benefit your organization?
4. What category describes your organization (tick all that apply):
   1. Not for Profit
   2. Health Services (including Health Authority staff)
   3. Health Professional (including doctors, nurses, care aids)
   4. Local Government Organization
   5. Provincial Government Organization
   6. Federal Government
   7. Academic Institution
   8. Funding Agency
   9. Private Industry
   10. Other ____________________________
5. What issues are you most interested in, when it comes to the CBSS (tick all that apply)?
   1. Transportation
   2. Wellness
   3. Recreation and Physical Activity
   4. Information, Referral, and Advocacy
   5. Nutritional Supports
   6. Housing

*Thank you for completing the survey – we look forward to your attendance and participation at the Summit.*

POST-SUMMIT SURVEY

# FOR SUMMIT ON AGING ATTENDEES

1. In a sentence or two, what was the most valuable ‘thing’ about attending the Summit? What stands out to you now?
2. After the Summit, did you start to view any of the issues you were interested in differently? If yes, please describe.
3. Did you start to do anything differently (in your organizations)?
4. Did you make any new connections at the Summit?
   - If yes, how many have you followed up on? Have you kept in touch with these people?
   - If yes, have you developed any collaborations or partnerships?
   - Please describe _________________________
5. Where is the sector going, what can you contribute and what can the sector do together?
6. What barriers do you foresee with the CBSS movement?
7. What should the next Summit focus on? In your mind, what are the next steps for the CBSS?
8. What category describes your organization (tick all that apply):
   - Not for Profit
   - Health Services (including Health Authority staff)
   - Health Professional (including doctors, nurses, care aids)
   - Local Government Organization
   - Provincial Government Organization
   - Federal Government
   - Academic Institution
   - Funding Agency
   - Private Industry
   - Other ____________________________
9. What issues are you most interested in, when it comes to the CBSS (tick all that apply)?
   - Transportation
   - Wellness
   - Recreation & Physical Activity
   - Information, referral, and Advocacy
   - Nutritional Supports
   - Housing

*Thank you for completing the survey.*

POST-SUMMIT Survey & Interview Guide

# FOR SUMMIT ON AGING ATTENDEES

Survey Questions

**preamble**

The purpose of the survey and interview is to gain an understanding of the Summit’s impact on your work in the community-based seniors’ service (CBSS) sector. There are four main sections within the interview: general questions and then questions focused on partnerships, resources, and issues. If we miss asking you something that you think is important – please ensure that you share it before the interview ends.

**Identification**

1. Input Participant Number [text field]

**General**

1. How would you categorize your organization?:

- Not for Profit
- Health Services (including Health Authority staff)
- Health Professional (including doctors, nurses, care aids)
- Local Government Organization
- Provincial Government Organization
- Federal Government
- Academic Institution
- Funding Agency
- Private Industry
- Other – [text field]

1. What’s your role within your organization?

- Leadership (executive director, director, manager)
- Staff (administrator, coordinator)
- Board or advisory committee member (including volunteer roles)
- Program volunteer
- Health professional (physician, nurse, care aide)
- Other – [text field]

1. What was the most valuable ‘thing’ about your participation in the Summit? Did it prompt any specific action?

**Partnership Focus**

1. Generally, do you believe that meaningful partnerships or collaborative networks are being achieved in your line of work?
   - If yes, what supports those? [text field]
   - If no, why not? Are there areas that can be improved? What supports or next steps need to be in place to facilitate this? [text field]
2. Did any new partnerships evolve from your participation in the Summit?

- If yes, what are your areas of mutual interest (i.e. specific services or issues)?
  - Have these connections prompted collaborative work?[text field]
  - How have you followed up on the connections (i.e. phone meetings, in-person planning sessions)? [text field]
  - How would you describe the quality of the connections (i.e. short-term connection to share information or long-term partner or collaboration project)? [text field]

1. In terms of future events, how do you want the CBSS to meet or connect in the future (i.e. Another Provincial Summit-type event? Other smaller scale events? Bigger events)? [text field]
   - What would you want the focus of the meeting to be (i.e. consultation, networking, knowledge sharing, or other focus)? [text field]

**Sector Specifics**

**As defined at the Summit, the categories of seniors’ services are: transportation; physical activity and recreation; information, referral, and advocacy; nutritional supports; wellness programs, education, and creative arts; affordable housing.**

1. From your perspective, how adequate are the seniors’ services in your community? Please share some insights on what specific services that are working well and not working well in your community.
   - What is required to build and strengthen CBSSs at the local level?
   - What resources do you need to move forward in your work supporting community-based seniors’ services?
   - [text field]
2. General, what do you see as the most important in building and strengthening CBSSs?
   - [text field]

**The Knowledge Hub will be an on-line and in-person learning network that will build on the expertise of people already working in the sector. It will be designed to strengthen BC’s Community Based Seniors' Services sector, increase organizational and sector capacity through information sharing, training, mentoring, communities of practice, policy development, and resource development.**

1. What do you know about the knowledge hub?
   - How likely are you to use the knowledge hub? [text field]
   - What is the value of the knowledge hub? [text field]
   - What are the barriers or opportunities to the idea of the knowledge hub? [text field]
2. Are there local planning tables that focus on seniors’ services operating in your area now?
   - If yes, tell me about how they formed, how they operate, and what you think is needed to enhance their functionality. [text field]
   - If no, tell me what would facilitate its creation? What are the barriers and facilitators to its development? What would their value be in your community? [text field]

Think about the key issues discussed at the Summit.

1. What are the key issues facing community-based senior’s services?
   - [text field]
2. Did you start to view any of the issues discussed at the Summit differently?
   - If so, what prompted the shift in perspective? [text field]
3. What role could an organization like yours play in the CBSS sector?
   - [text field]
4. What could someone in a role like yours do to support the CBSS sector?
   - [text field]

The scope of the work and practice of CBSSs have expanded – more and more they are looked to as health promotion spaces.

1. How can CBSSs respond to this expectation to become better positioned to serve as health promotion spaces for seniors?
   - [text field]
   - What are the challenges and opportunities for CBSS organizations as they take on these health promotion roles?
   - [text field]
2. More and more, CBSSs are offering physical activity focused programming
   - What needs to be in place to do this well?
   - What are the challenges in offering physical activity focused programming?
   - What are opportunities or assets that can be strengthened to do this well?

Interview Questions

**Interview Instructions**

- **Review the participant’s survey responses before interviewing; refer to their responses to dig deeper into aspects of the questions in this guide.**
- **Acquire iPhone and ensure it is charged; book private office with a phone with speaker capability.**
- **Before recording inform the participant of what to expect over the interview – offer the preamble.**
- **Remind them that the interview will take ~20 minutes, that the interview will be recorded and that transcriptions of the interview will be de-identified before analysis. All individual response will be kept confidential and only summarized data will be shared.**
- **Provide the preamble script.**
- **Begin recording.**
- **State study name.**
- **State date and interviewer name.**
- **State participant number.**

**preamble**

**The purpose of the survey + interview is to gain an understanding of the Summit’s impact on your work in the community-based seniors’ service (CBSS) sector. If we miss asking you something that you think is important, please share it before the interview ends.**

**General**

1. What was the most valuable ‘thing’ about your participation in the Summit? Did it prompt any specific action?

**Partnerships**

**Only ask these questions if they note in the survey that partnerships/collaborations developed from the Summit. If not, just quickly confirm that partnerships were not a key impact/output from their Summit experience.**

1. Tell me about any partnerships and collaboration that were initiated by your participation at the Summit?

- What are your areas of mutual interest (i.e. specific services or issues)?
- Have these connections prompted collaborative work?
- How would you describe the quality of the connections?

1. How do you want the CBSS sector to meet or connect in the future? What should the focus be?

- Another Provincial Summit-type event? Other smaller scale events? Bigger events?
- Do you want the focus of the events to be on consultation, networking, knowledge sharing, or other focus?

**Sector Specifics**

**Let’s expand on your responses in your survey:**

1. General, what do you see as the most important in building and strengthening CBSSs?
2. What do you know about the knowledge hub? What is the value of the knowledge hub?
   - How likely are you to use the knowledge hub?
   - What are the barriers or opportunities to the idea of the knowledge hub?
3. In your local area, are there local planning tables or communities of practice that focus on seniors’ services?
   - If yes, tell me more about them….
   - If no, tell me what would facilitate their creation? What are the barriers and facilitators to its development? What would their value be in your community?
4. How can CBSSs respond to this expectation to become better positioned to serve as health promotion spaces for seniors?
   - What are the challenges and opportunities for CBSS organizations as they take on these health promotion roles?
5. More and more, CBSSs are offering physical activity focused programming

- What needs to be in place to do this well?
- What are the challenges in offering physical activity focused programming?
- What are opportunities or assets that can be strengthened to do this well?

**Conclusion**

1. Is there something from your responses on the survey that you wanted to expand on, that we have not yet covered?
2. That’s all of the questions. Do you have anything else to add before we conclude our interview?

**Thank you for your time.**
